# Illuminating the pathological, immunohistochemical, and molecular investigations of myelocytomatosis in chicken embryos

**DOI:** 10.1186/s13027-026-00731-0

**Published:** 2026-02-02

**Authors:** Ahmed Fotouh, Ahmed K. Elsayed, Ali Mahmood Zanaty, Said Elshafae, Eman Abd El‑Menamm Shosha

**Affiliations:** 1 Department of Pathology and Clinical Pathology, Faculty of Veterinary Medicine, New Valley University, Kharga, Egypt; 2 Department of Anatomy and Embryology, Faculty of Veterinary Medicine, Suez Canal University, Ismailia, 41522 Egypt; 3 Gene Analysis Unit, Reference Laboratory for Quality Control on Poultry, Animal Health Institute, Agriculture Research Center (ARC), Giza, Egypt; 4 Department of Pathology, Faculty of Veterinary Medicine, Benha University, Moshtohor, Toukh, Qaluiobiya 13736 Egypt; 5 Department of Pediatrics, College of Medicine, University of Utah, Salt Lake City, UT USA; 6 Virology Department, Faculty of Veterinary Medicine, New Valley University, Kharga, Egypt

**Keywords:** Myelocytomatosis, Avian leukosis virus, PCR, Chicken embryos, Immunohistochemistry, Sequencing

## Abstract

*Avian leukosis virus* Subgroup-J (ALV-J) is an avian retrovirus affecting a wide range of avian species, and has posed a great threat to the local poultry with significant losses. In Egypt, ALV-J has been actively monitored in various breeder flocks. This study investigated 200 eggs from ALV-J positive breeders, confirmed positive-polymerase chain reaction (PCR)-ALV-J, collected from a hatchery between January 2022 and December 2023. 200 tissue specimens (liver, heart, spleen, kidney, and lung) were collected from suspected embryos for PCR test, immunopathological identification, and sequencing analysis. To the best of our understanding, this is the first study specifically detecting ALV-J infection in chicken embryos in Egypt. The current study focuses on the genetic evolution and sequencing analysis of ALV-J isolates from chicken embryos across three Egyptian governorates: El-Sharkia, Al-Qalyubiya, and New Valley. Postmortem findings of the infected embryos showed stunting, curling, dwarfing, hemorrhagic body surface, enlarged liver, and congestion of chorio-allantoic membranes (CAM). Histopathological observation demonstrated no lymphoid or myeloid cell infiltrations, only degenerative changes and congestion of the examined organs existed. The immunohistochemical staining confirmed that the viral-positive signals were visceral tissues as the spleen, liver, and kidney. Only 30 samples were PCR-positive for ALV-J *gp85* gene at size of 545 base pairs with a prevalence rate of 15%. Two ALV-J positive samples were sequenced and deposited in the Genbank under accession numbers (PQ119499 - PQ119500, ALV-J-II). ALV-J-*gp85* gene phylogeny showed that the AlQalyubiya-1-EGYALVJ-env isolate is highly genetically correlated to Chinese strains (KJ179914-ALV-J-GD27, KJ179912-ALV-J-GD19, KJ179911-ALV-J-GD31) with nucleotide and amino acid identities of 98–100%; respectively. Moreover, Newvalley-2-EGYALVJ-env isolate is close similar to previous mentioned Chinese strains with nucleotide identity percentages of 98% and amino acid identity percentage 97%, 96%, 96%; respectively. This study confirmed that our two ALV-J isolates are not completely similar to the current Egyptian isolates. Also, ALV-J infection was continuously distributed in the breeders and considered one of the factors contributing to the tumor epidemics.

## Introduction

Avian oncogenic viruses can cause significant tumors or cancer-like growths in birds, including *Marek’s disease virus* (MDV), *Avian leukosis virus* (ALV), and *Reticuloendotheliosis virus* (REV). These viruses pose serious economic threats to the poultry industry due to reduced productivity, increased mortalities, and control costs [1]. ALV, an oncogenic retrovirus, belongs to the genus *Alpharetrovirus* in the family *Retroviridae*. It is known to cause a range of neoplastic diseases, immunosuppression, and reduced fertility in various avian species worldwide [2, 3]. In the 1980s, broiler breeders and meat-type hens in the United Kingdom produced HPRS-103, the first strain of ALV subgroup J (ALV-J) to be identified and isolated [2, 4]. Furthermore, the first reports of ALV-J infection in China were reported in 1999 in broiler flocks, which led to devastation to native breed layers and wild ducks, causing serious economic losses [5]. Based on host range, viral envelope composition, and cross-neutralization patterns, ALVs are currently classified into 11 subgroups [5]. Subgroups A, B, C, D, E, K, and J, which primarily infect chickens and turkeys, are categorized as exogenous viruses. Subgroup E, on the other hand, is an endogenous virus. Indeed, ALV-A and ALV-B are particularly notable for their role in causing lymphoid leukosis with high incidence rates [6], while subgroups ALV-C and ALV-D infections in chickens are rare [7]. More recently, ALV-K has been linked to the induction of fowl glioma [8].

Importantly, ALV-J was recognized as the furthermost predominant and economically significant strain in the poultry sector [4]. Chickens primarily infected with ALV-J, also known as avian leukosis myelocytomatosis-associated virus, typically exhibit multiple systemic tumors, leukemic infiltrates in the bone marrow and other organs, and immunosuppressive syndromes that lead to secondary bacterial infections, such as those caused by *E. coli* and *Salmonella spp* [9, 10]. This infection also results in growth hindrance, significantly reduced farm production, and a substantial increase in losses, especially in breeder flocks. Additionally, Myeloid leukosis (also known as myelocytomatosis) is specifically linked to ALV-J infection in breeder hens [11]. Obviously, abundant cases of myelocytomatosis in broiler breeders have also been reported in Europe [12], Australia [13], the Americas, Asia [14], and Egypt [15]. The tumor cells associated with myelocytomatosis originate from the myeloid lineage, including immature precursor cells called myeloblasts and other granulocytic cells.

ALV-J is capable of both horizontal and vertical transmission, leading to significant global impacts on the poultry industry, as infected chickens often develop multiple tumor phenotypes and exhibit reduced weight gain. The vertical transmission can occur from infected parents to their progeny through the eggs [16, 17]. Nevertheless, crossbreeding local breeds with other breeds may increase the incidence of ALV-J infection, particularly through vertical transmission [18].

Notably, ALV-J transmission to embryos can happen via two primary mechanisms: first, through inherited loci encoding endogenous ALV-J, which depends on the endogenous virus loci and the host genome; and second, by the shedding of ALV-J from the gonads and oviducts of infected birds [19, 20]. Additionally, ALV-J can integrate its genome into the DNA of reproductive cells of the hen or rooster, leading to its incorporation into the DNA of the germ cells; attributing integration in the embryogenome. As the embryo develops, ALV-J can replicate and spread to various tissues of the developing embryo, including bone marrow, liver, spleen, and other organs. In particular, ALV-J infection in congenitally infected embryos may reduce the hatchability rates, as the developed embryos fail to grow properly or may die before hatching. ALV-J-infected embryos can result in decreased fertility and hatchery performance in affected flocks [21–23]. In Egypt, ALV-J-induced myelocytomatosis has been identified in broiler flocks through gross pathology, histopathological examination, serology, molecular characterization, and sequencing analysis [15, 24]. In layers, ALV-J is predominantly found in the ovarian stroma, particularly in bud cells in contact with oogonia, and oocytes, and is highly concentrated in albumen-secreting glands. Exposure of the ovaries and oviducts can lead to early and diffuse viral infection [23, 25].

Due to no available vaccination programs, so the control measures should be focused on the removal of infected breeders, implementation of management strategies, selection of ALV-J-resistant breeding stock, and stringent biosecurity protocols on poultry farms to minimize ALV-J transmission from infected parents [26]. Thus, rapid and accurate diagnosis is critical for effectively managing and eradicating ALV-J from breeding flocks [27]. It is noteworthy to state that molecular diagnostic techniques, such as PCR and sequencing analysis, are crucial for understanding the genetic diversity, pathogenesis, and epidemiology of ALV-J [28]. Additionally, the ALV genome includes gag, pol, and env as crucial structural proteins, which have translated into the specific-group antigen and envelope glycoproteins. The ALV genome encodes the *gp85* gene, a key envelope glycoprotein, is the most variable and rapidly evolving region in ALV-J [26, 29, 30]. The *gp8* gene is closely involved in viral entry, inducing neutralizing antibodies, tumor formation, tissue tropism, and virulence [31–33]. Besides, pathological and immunohistochemical examination of ALV-J-induced myelocytomatosis in chicken embryos helps to characterize the nature and extent of tumor formation, impact on affected tissues, as well as provides insights into the disease process.

This is the first specified study in Egypt reported on ALV-J with myelocytomatosis in chicken embryos. Using PCR testing and molecular sequencing, this study focuses on the genetic characterization and sequencing of ALV-J isolates from chicken embryos in the Egyptian governorates of El-Sharkia, Al-Qalyubiya, and New Valley. Additionally, the study provides a comprehensive diagnosis of myelocytomatosis in naturally infected embryos, including a full pathological examination and immunohistochemical staining of affected internal organs.

## Materials and methods

### Ethical statement

This study protocol was achieved by ensuring ethics and rules for experimental animals and approved by the New Valley Research Ethics Committee of the faculty of veterinary medicine, New Valley University, under number (02/3/6–2024/19).

### Clinical background and specimen assembly

Two hundred eggs collected from ALV-J-infected breeders, confirmed previously by histopathology and PCR, were investigated at the hatchery during the period from January 2022 to December 2023. The embryos’ age ranged from 16 to 18 days. The hatchery was located in three Egyptian governorates: El-Sharkia, Al-Qalyubiya, and New Valley. Collectively, a total of 200 liver and heart specimens were collected from suspected diseased embryos for PCR tests. Additionally, the ALV-J-positive breeder flocks exhibited general manifestations such as depression, anorexia, and tumors.

### Microscopic analysis

Liver, spleen, kidney, lung, and heart tissue samples were collected from chicken embryos for histological examination. After being washed, dehydrated, cleared, and embedded in paraffin, the specimens were fixed with 10% neutral buffered formalin. Paraffin-implanted tissue blocks were then segmented at a thickness of 4 μm and stained with hematoxylin and eosin (H&E) for minute investigation [34].

### Immunohistochemistry examination (IHC)

To recognize the presence of ALV-J antigen, the paraffin-embedded segments were put on certain glass slides at a thickness of 4 μm. The segments were handled utilizing a standard monotonous streptavidin-biotin/horseradish peroxidase (HRP). Pre-treatment included brooding with 3% H_2_O_2_ in methanol, trailed by hindering with 5% BSA (cow-like serum egg whites) in phosphate-supported saline (PBS) for 10 min. After that, a primary antibody (rabbit anti-ALV-A surface protein, Santa Cruz, CA, USA, can detect ALV-J and other subgroup viruses) and a secondary antibody (biotinylated goat anti-rabbit IgG, Santa Cruz, CA, USA) were used to incubate the slides. Streptavidin/HRP was applied after several washes. The neutralizer restricting destinations were imagined utilizing 3,3’- diaminobenzidine (DAB) as the chromogen, bringing about a dull earthy colored encourage (positive staining). Counterstaining was done with hematoxylin. Positive and negative control slides were given by the pathology division, Creature Wellbeing Establishment, Dokki, Giza, Egypt. The slides were then inspected under a light magnifying instrument [35].

### Genome extraction and PCR analysis

Particularly, liver samples were stored at -70 °C until subsequently utilized for molecular detection of ALV-J through PCR test following the manufacturer’s recommendations. Genomic DNA extraction followed the strategy depicted by Murray and Thompson [36] and Smith et al. [37]. A conserved part of the gp85 gene associated with ALV-J was specifically targeted by a pair of oligonucleotide primers, the forward primer H5 and the reverse primer H7, in the PCR assay. 545 base pairs (bp) of PCR amplicon product are produced by this primer set. The preliminaries were planned according to the ALV-J prototype strain (HPRS-103) (accession number: Z46390) (Table 1).

**Table 1:** Sequence of specific primer sets, and PCR amplicon product sizes to amplify *gp85* gene of ALV-J

Primer for ALV-J	Sequence (5’–3’)	Product size (bp)	Reference
H5-Forward	5’-GGATGAGGTGACTAAGAAAG-3’	545	[37]
H7-Reverse	5’-CGAACCAAAGGTAACACACG-3’		

### Sequencing, phylogenetic, and recombination analysis

After being thoroughly purified with the QIAquick Gel Purification Kit (Qiagen, Hilden, Germany), the two selected positive amplicons were sequenced with the BigDye Terminator v3.1 Cycle Sequencing Kit (Applied Biosystems, California, USA) on an ABI 3500 Genetic Analyzer (Life Technologies, California, USA) with specific primers that targeted the ALV-J gp85 gene. the Clustal W program was used to align the inferred sequences with other correlated strains deposited in GenBank. Also, a phylogeny designed tree was conducted using the MEGA-X [38] and BioEdit software, with levels of 1,000 bootstrap replicates [39]. Recombination analysis of the ALV-J strain was investigated following the method of the RDP-5 Program [40]. The used algorithms, including BootScan, MaxChi, GENECONV, SiScan, Chimaera, LARD, RDP5, Phyl-Pro, and 3Seq, were used for accurate comparison [41]. Recombination analysis was carried out by four or more independent procedures, regarded as clear positive occurrences.

## Results

### Clinical and gross findings

**Fig. 1:**
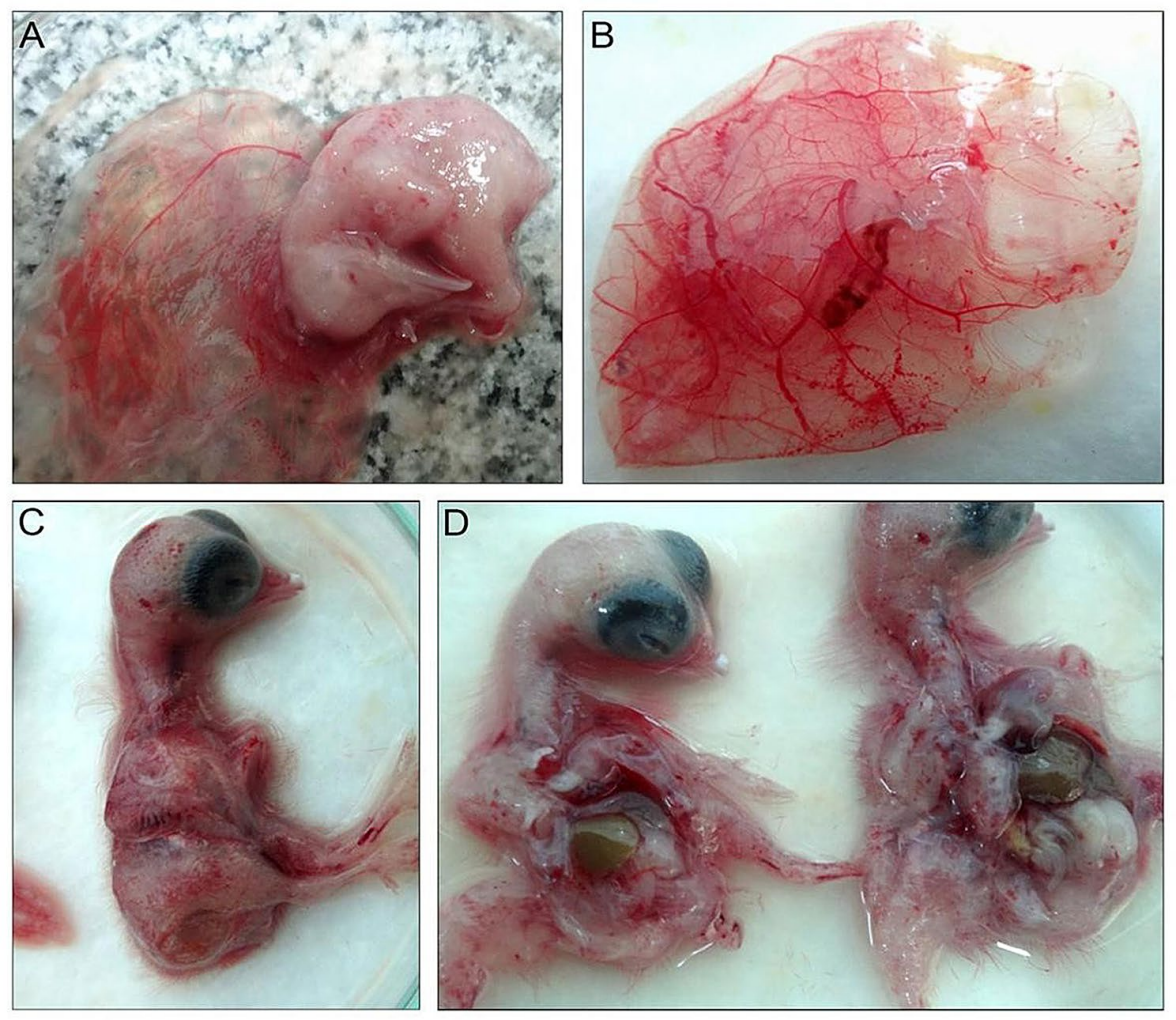
Different chicken embryos showing: **A**) stunting, curling, and dwarfing anomalies. **B**) congestion of the chorioallantoic membrane (CAM). **C**) hemorrhages on the body surface. **D**) enlarged livers

Regarding eggs external appearance, ALV-J-positive eggs are apparently normal without any softness or small size. Clinical signs in the breeder flocks at El-Sharqia, Al-Qalyubiyya, and New Valley governorates were mostly non-specific, including paralysis, depression, dehydration, weakness, anorexia, and growth retardation, with a recorded mortality rate of 7%; meanwhile, the morbidity reached 20%. Postmortem findings showed stunted, curled, dwarfed embryos with hemorrhages on their body surface, enlarged embryonic liver, and congested chorio-allantoic membranes (CAM) (Fig. 1**).**

### Microscopic alterations and immunohistochemical reactions

**Fig. 2:**
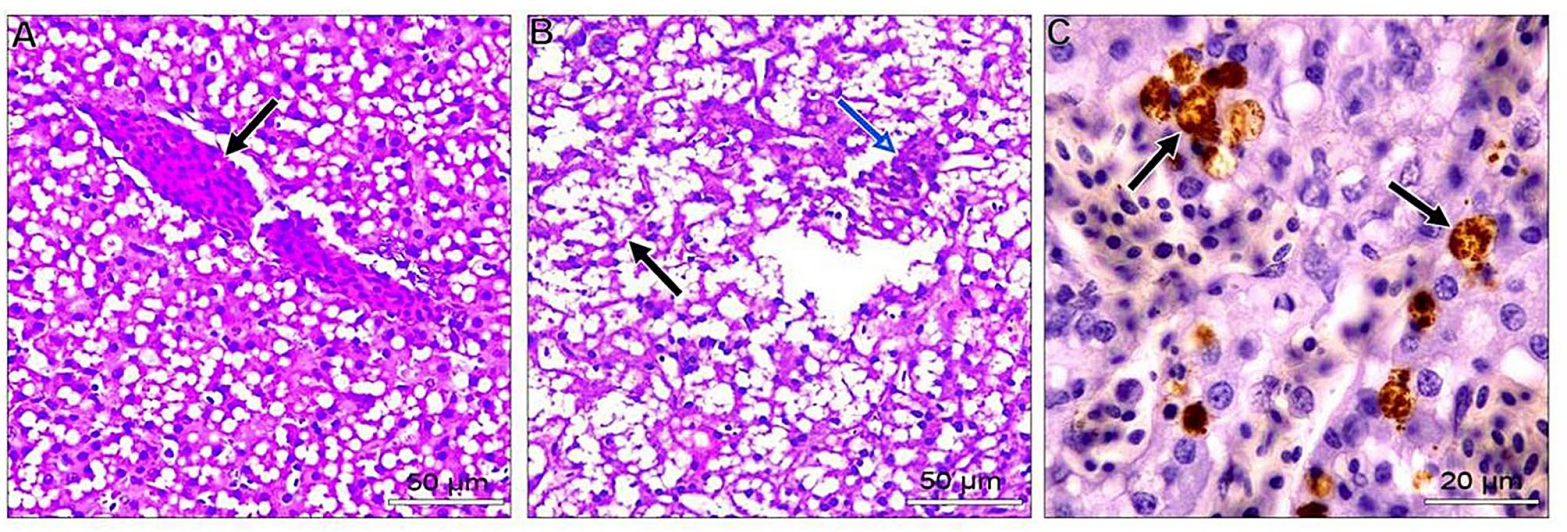
Embryo’s liver; **A**) showed congestion and dilatation of the central vein (arrow) (H&E, scale bar: 50 μm). **B**) proliferation of reticular cells (black arrow) and nuclear pyknosis (blue arrow) (H&E, scale bar: 50 μm). **C**) positive viral antigen reaction represented by brownish coloration in some hepatocytes (arrows) (IHC scale bar 20 μm)

**Fig. 3:**
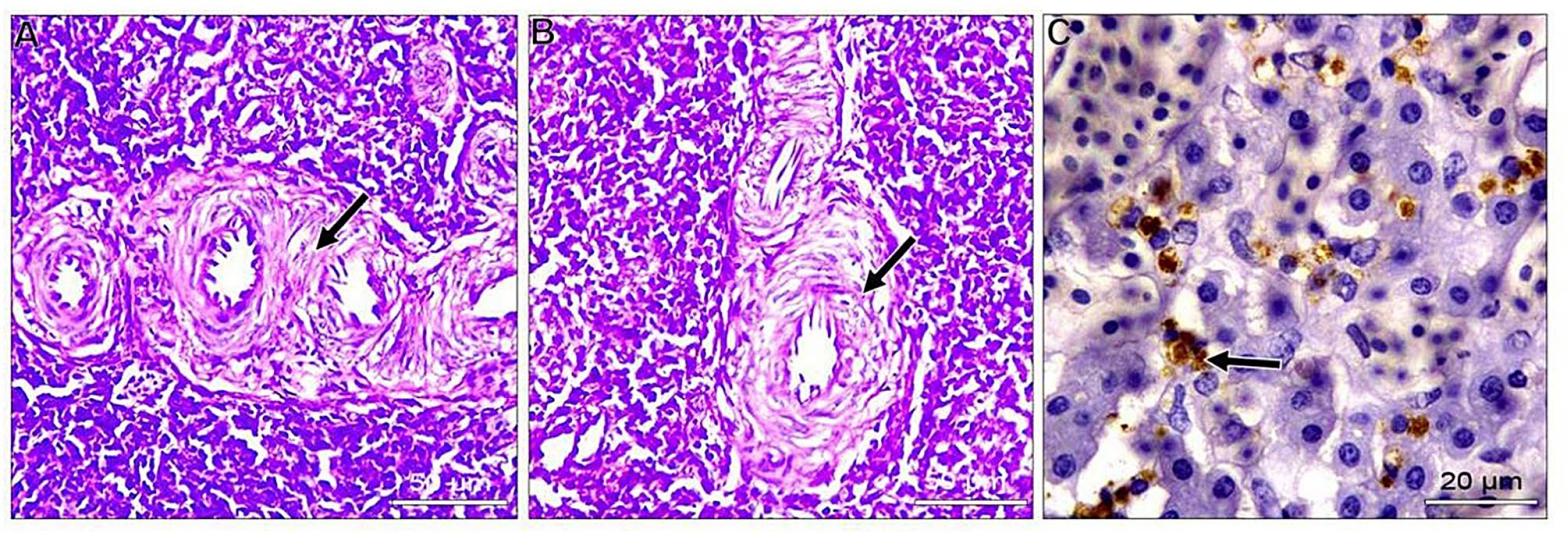
Embryo’s spleen; **A** and **B**) severe degeneration of the tunica media of splenic arterioles, hypertrophy, and cytoplasmic vacuolation. (arrow) (H&E, scale bar: 50 μm). **C**) positive viral antigen reaction represented by brownish coloration in some lymphocytes (arrows) (IHC scale bar 20 μm)

**Fig. 4:**
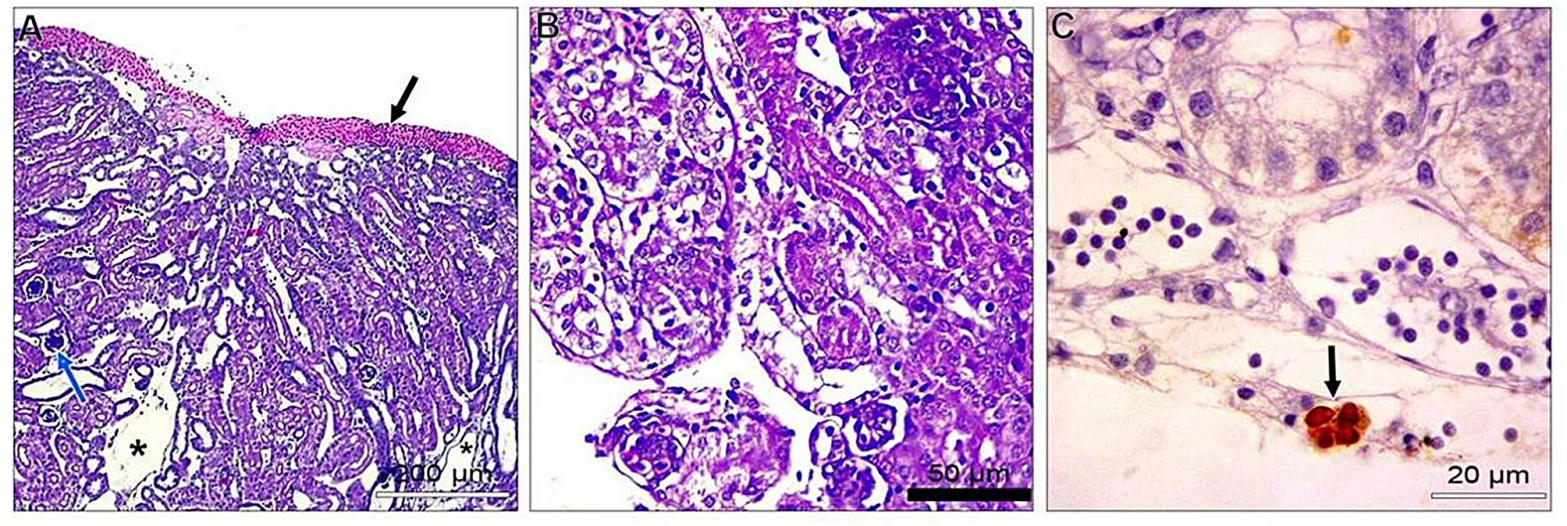
Embryo’s kidneys; **A**) showed diffuse hemorrhage in subcapsular (black arrow) and atrophied glomerulus (blue arrow) (H&E, scale bar: 200 μm). **B**) vacuolar degeneration of convoluted tubules (H&E, scale bar: 50 μm). **C**) positive viral antigen reaction represented by brownish coloration (arrows) (IHC scale bar 20 μm)

**Fig. 5:**
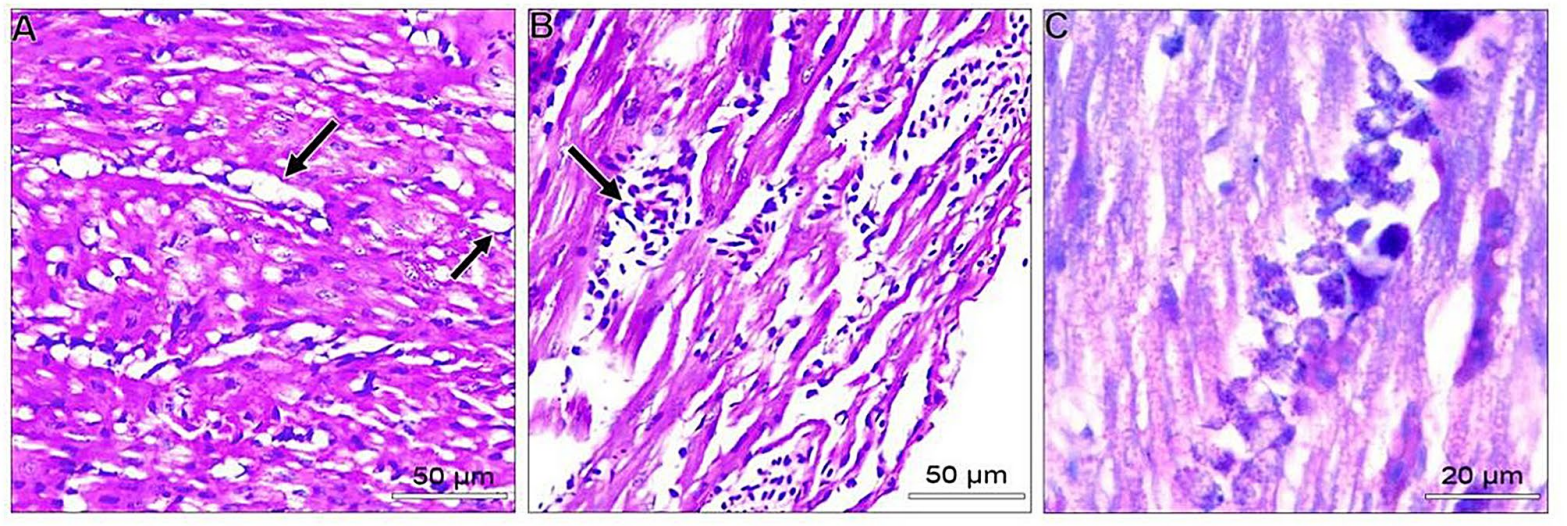
Embryo’s heart; **A**) showed vacuolation of myofibers (arrows) (H&E, scale bar: 50 μm). **B**) infiltration of mononuclear inflammatory cells (arrow) (H&E, scale bar: 50 μm). **C**) No viral antigen reaction was detected (IHC scale bar 20 μm)

**Fig. 6:**
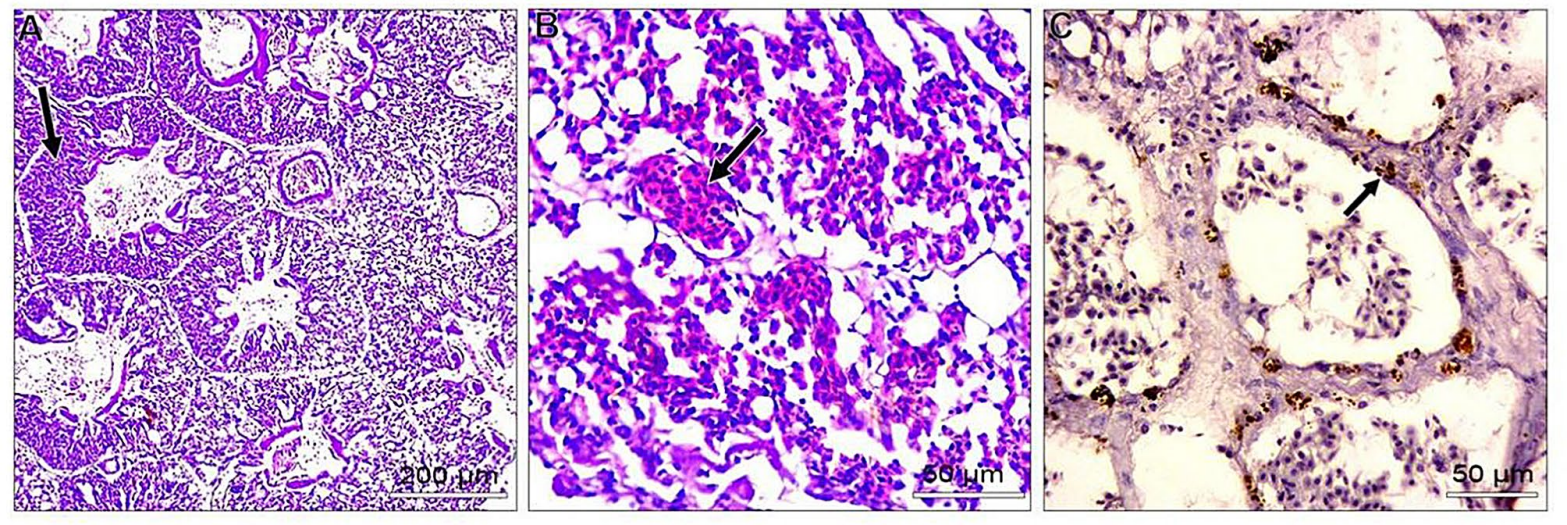
Embryo’s lung; **A**) showed thickening of the wall of bronchioles due to infiltration by mononuclear cells (arrow) (H&E, scale bar: 200 μm). **B**) Congestion of interstitial blood vessels and may be hemorrhages (arrow) (H&E, scale bar: 50 μm). **C**) positive viral antigen reaction represented by brownish coloration in some alveolar lining epithelium (arrows) (IHC scale bar 20 μm)

In the liver, most examined tissues showed vacuolar degeneration to different degrees. Mild congestion of hepatic blood vessels, including central veins and sinusoids. Focal areas of small hemorrhages may be detected within hepatic parenchyma. Some livers suffered from the proliferation of reticular cells and loss of tissue architecture. Areas of nuclear pyknosis and karyorrhexis were seen. By immunohistochemical staining, viral antigen represented by brown staining was detected in some hepatocytes and Kupffer cells **(**Fig. 2A-C**).** Concerning the spleen, the most consistent lesion of splenic tissue was severe degeneration of splenic arterioles, especially the tunica media. This layer showed hypertrophy and cytoplasmic vacuolation. Some splenic parenchyma showed an increase in the number of splenic arterioles. Regarding to immunohistochemical reaction, the viral antigen represented by brown staining was detected in lymphocytes (Fig. 3A-C). Regarding kidneys, minute hemorrhages in renal interstitial tissues and, in some cases, diffuse hemorrhages were detected, especially under the renal capsule. Hypercellularity of the glomerulus may be atrophied. Convoluted tubules showed mild vacuolar degeneration with pyknosis of many nuclei. Some cases showed interstitial edema. For viral antigen distribution, a mild positive reaction was observed in the convoluted tubules epithelium and in the capsule (Fig. 4A-C). Also, in the heart, disorganization of myofibers with vacuolation of cytoplasm was detected. In some myocardial tissues, there was mononuclear cellular infiltration and interstitial edema. Minute hemorrhages were also detected. No immunohistochemical reactions were observed for the specific viral antigen (Fig. 5A-C). Subsequently in the lungs, thickening of the wall of bronchioles due to infiltration by mononuclear cells and hyperplastic epithelium. Desquamated epithelium and debris may be detected in the lumen of some bronchioles. Congestion of interstitial blood vessels and may be hemorrhages that compress the alveoli. Positive viral reactions were mainly detected in alveolar lining epithelium (Fig. 6A-C).

### Genomic characterization

**Fig. 7:**
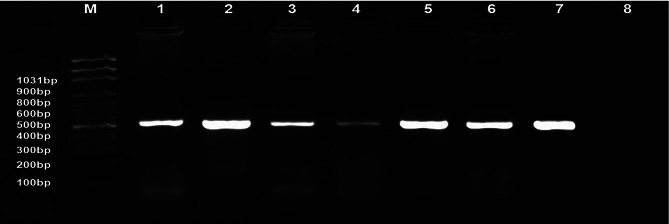
Molecular identification by polymerase chain reaction assay for ALV-J-gp85 gene-positive isolates using ALV primer. Lanes 1–6 are positive samples at 545 bp; Lane 7 is a positive control, Lane 8 is a negative control; M: represents a 100-bp ladder as a size standard

A *gp85-*PCR amplicon of ALV-J was genetically characterized at 545 bp. Collectively, 200 liver and heart samples were examined from ALV-J-infected embryos, and 30 positive samples were confirmed positive with a prevalence rate of 15% (Fig. 7). Additionally, there are no other common avian diseases screening or other subgroups of ALV-J as this study mostly focus on the ALV-J vertical transmission from breeders to their progeny.

### ALV-J partial sequencing, phylogenetic characterization

**Fig. 8:**
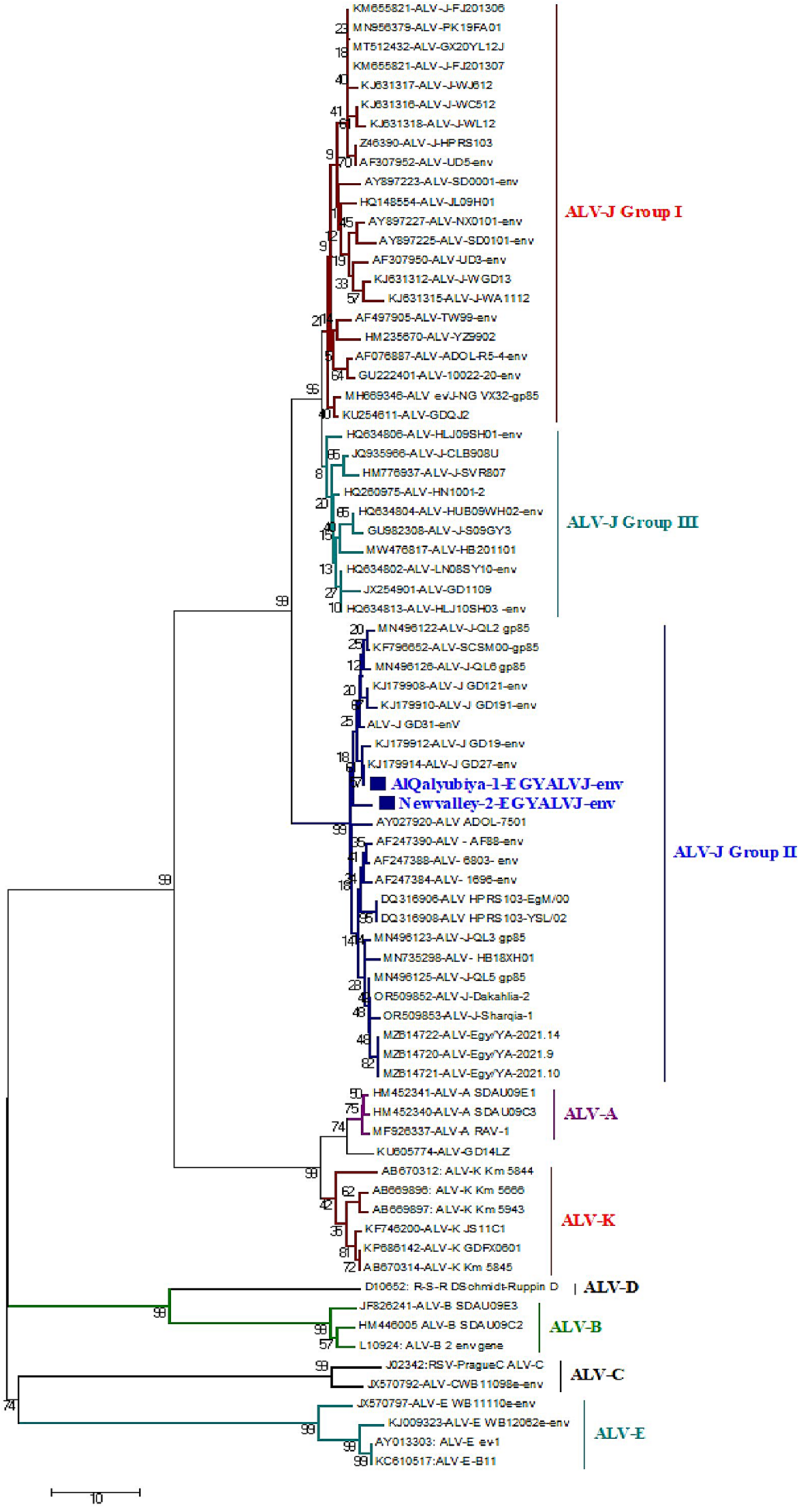
Phylogenetic tree corresponding to ALV-J-gp85 gene sequence alignments with other reference Genbank strains. The phylogenetic analysis reported that the AlQalyubiya-1-EGYALVJ-env isolate and Newvalley-2-EGYALVJ-env isolate, located with Egyptian and Chinese strains, clustered in subgroup II. Both isolates are indicated by a square. The tree was constructed using the neighbor-joining method with 1,000 bootstrap replicates, using Molecular Evolutionary Genetics Analysis 7.0

Phylogenetic analysis was conducted including 75 defined Genbank strains of other ALV subgroups. Notably, the phylogenetic trees corresponding to *gp85* gene revealed that all referred ALV-J isolates (AlQalyubiya-1-EGYALVJ-env and Newvalley-2-EGYALVJ-env) clustered as subgroup II with accession numbers PQ119499 - PQ119500 comparable to other referential strains of HPRS103 and ADOL-7501 (Fig. 8). AlQalyubiya-1-EGYALVJ-env isolate showed genetic similarity to contemporary Chinese strains (ALV-J-GD27, ALV-J-GD19, ALV-J-GD31) with nucleotide identity of 99–100% and the amino acid identity was 98–100%, respectively. This high similarity may suggest common ancestry. Likewise, the AlQalyubiya-1-EGYALVJ-env isolate shared 87–95% nucleotide and amino acid identities with ALV-Egy/YA-2021.9, ALV-Egy/YA-2021.14, ALV-J-Dakahlia-2, ALV-J-Sharqia-1, ALV-J-QL5 gp85 (Egyptian isolates, clustered as ALV-J group II), ALJ-ADOL-7501 (American reference strain, clustered as ALV-J group II); respectively (Table 2). Likewise, Newvalley-2-EGYALVJ-env isolate has the highest nucleotide homology (98%) to previously mentioned Chinese strains and amino acid homology of 96–97%, respectively. Moreover, Newvalley-2-EGYALVJ-env isolate shared nucleotide identity of 92%, 92%, 90%, 92%, 87%, 94%; respectively with ALV-Egy/YA-2021.9, ALV-Egy/YA-2021.14, ALV-J-Dakahlia-2, ALV-J-Sharqia-1, ALV-J-QL5 gp85, ALJ-ADOL-7501, and based on the amino acid identity, the percentages were 94%, 94%, 92%, 93%, 91%, 93%; respectively. In addition, AlQalyubiya-1-EGYALVJ-env and Newvalley-2-EGYALVJ-env isolates have low homology with ALV-ev/J-NG_VX32 (Nigerian strain, ALV-J-I), ALV-J-FJ201307(Chinese strain, ALV-J-I), ALV-UD3-env and ALV-ADOL-R5-4-env (American strains, ALV-J-I), ALV-J-HPRS103-EgM/00 **(**Egyptian strain, ALV-J-II), ALV-J-SVR807 (Russian strain, ALV-J-III), GD1109 (Chinese strain, ALV-J-III), with a percentage of (76%,75%), (75%, 74%), (79%, 80%), (89%, 88%), (81%, 80%), (82%, 81%); respectively (Table 2**)**. Comparative *gp85* gene analysis revealed 97–98% amino acid and nucleotide similarity between the two recently sequenced isolates themselves, indicating their close genetic relationship and probably having the same origin. In accordance of the RDP5 software analysis, the recombination assessment corresponding to the ALV-J-*gp85* gene of both isolates confirms no recombination sites evidence was demonstrated when compared with the ALV-J original HPRS-103 strain.

**Table 2:**
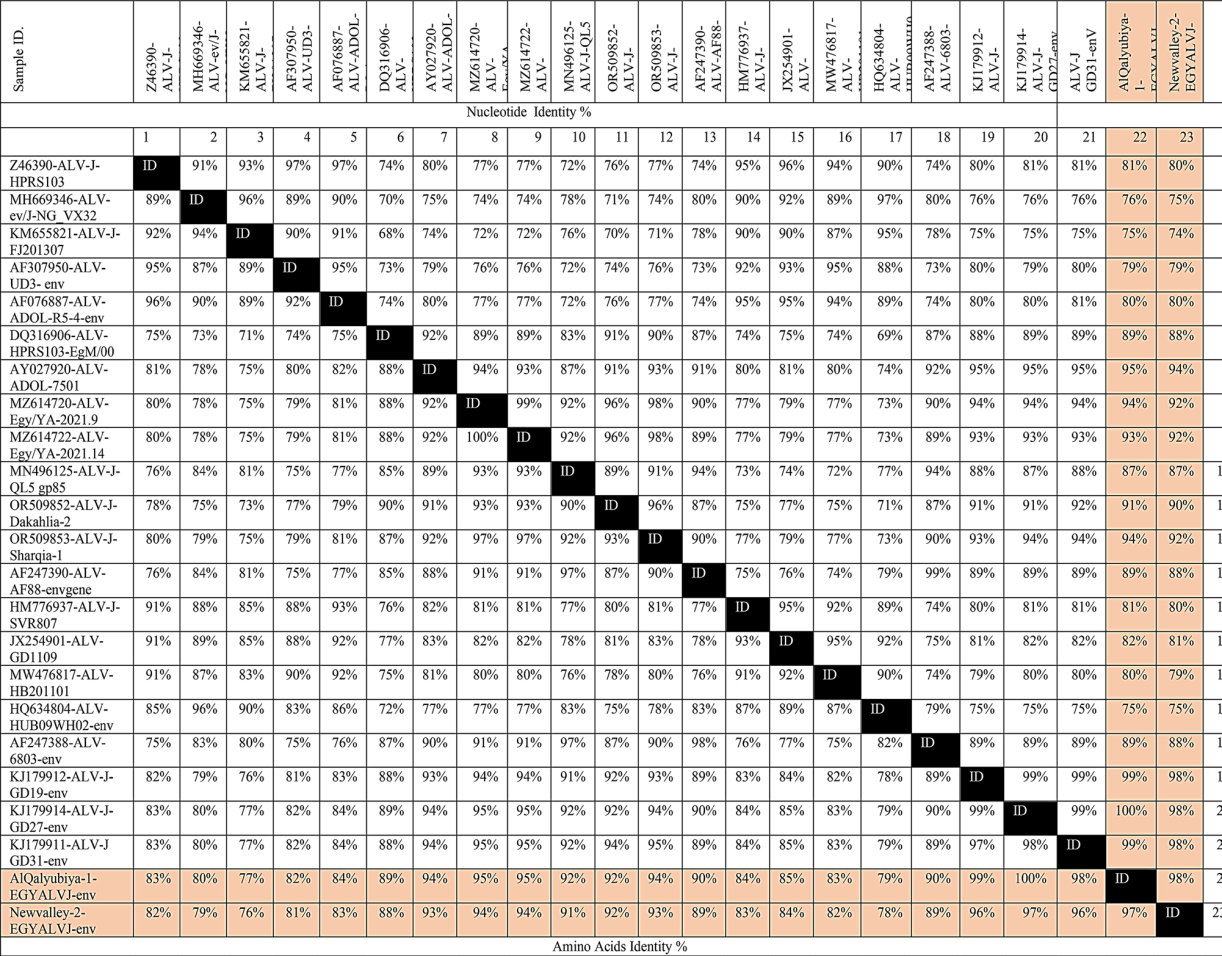
Nucleotide and amino acid identities of both ALV-J isolates in comparison to other reference strains

Amino Acids Identity %

Nucleotide and amino acids homology of ALV-J isolates in comparison to reference strains from China, Egypt, Russia, the United Kingdom, and the USA. The figure utilizes a comparative alignment in which the nucleotide identity varied from 74–100%, as well as the amino acids identity was 76–100%

## Discussion

The rise of various viral infections has become a growing concern, leading to significant epidemics that poses a major risk to the health of animals and poultry [42–47]. generally, these viral epidemics are attributed to RNA viruses, leading to devastating economic damages to poultry farming [48–50]. Avian immunosuppressive retroviruses, especially ALV-J, were a serious harm, causing significant economic losses in the fowl farms. More importantly, the ALV-J rapid spread and its novel strains emergence among poultry worldwide have led to the urgent need for viral eradication in commercial breeders [10, 24]. Infection control is difficult in the absence of a vaccination program, so the flock elimination is the only available control method to control its rapid spreading [24]. In China, ALV-J infection was successfully controlled by carefully selecting non-infected breeders [41, 51, 52]. In 2014, in Egypt, ALV-J rapidly spread throughout poultry flocks, affecting layers and ducks, resulting in high mortality rates [53]. At present, ALV-J with various genetic features has been detected in Egyptian poultry farms with subsequent molecular confirmatory approaches [34, 54].

The positive detection in breeders at El-Sharqia, Al-Qalyubiyya, and New Valley governorates displayed general clinical symptoms with morbidity and mortality rates of 20% and 7%. Concerning gross lesions of chicken embryos, there was evidence of ALV-J infection characterized by an enlarged liver, severe hemorrhagic body surface as well as stunting, curling, congested chorioallantoic membrane (CAM), and embryonic death within four days; attributing to the ALV-J direct effect. These necropsy results came in accordance with [34, 55].

ALV-J is considered as a tumor virus, so its confirmatory diagnosis depends mainly on histopathological investigation and PCR detection with sequencing analysis. The histopathological investigation, as well as genetic characterization, provides a precise diagnostic approach for ALV-J associated with myelocytomatosis [56, 57]. Regarding microscopic changes in all examined organs, there were no neoplastic cell infiltrations, either lymphoid or myeloid. This may be attributed to the nature of these neoplastic diseases. However, in avian lymphoid leukosis, the transformation of host cells generally requires more than five months, as multiple rounds of viral replication are necessary before a provirus integrates near a cellular proto-oncogene, resulting in its activation. Vertical transmission of ALV-J enables insertion of the viral genome into the DNA of germ cells, thereby facilitating its integration into the genome of the developing embryo [58]. Once the embryo develops from the fertilized egg, the ALV-J can replicate, inducing infection. Infected embryo’s organs suffered from degenerative changes as vacuolation of cells, congestion, hemorrhages, and mononuclear cells infiltration. The direct effect of ALV-J may be the main cause for these alterations. The same findings were recorded by [25, 59, 60]. In particular, antigen staining by IHC, the brown granules indicated the positive reaction for the presence of specific virions in the hepatocytes and Kupffer cells of the liver, lymphocytes of the white pulp of the spleen, lining epithelium of alveoli, as well as lining epithelium of convoluted tubules in the kidneys. These results support the previous findings [61–63].

Particularly, the PCR test with ALV-J-specific primers established 30 positive samples with a prevalence rate of 15%. This molecular pattern is similar to that of those [22] who recorded ALV infection, molecularly confirmed by the RT-PCR assay, in commercial eggs at 14.2%. Our finding underscores that ALV-J is the main etiology of viral tumors in broilers in Egyptian governorates (El-Sharkia, Al-Qalyubiya, and New Valley), thus no ALV other subgroups were tested. Due to horizontal and vertical transmission, ALV-J has caused increasingly severe damage to the poultry industry worldwide including Egypt (broiler breeder, layers, broiler) as infected birds exhibit various tumor phenotypes with decreased weight gain [17, 24]. Based on PCR data, the liver currently exhibits a high tropism for ALV-J. These findings are in line with those of [64], who observed a significant ALV-J tropism in the liver. Also, this finding suggested that the samples were gathered in the viremic stage. In line with our findings [15] assessed that all liver, spleen, and kidney samples showed a positive result with ALV-J at an amplicon size of 545 bp. In reference to our findings [26, 54], stated multiple ALV-J infections in various Lower Egypt governorates, as determined by qRT-PCR in breeders. According to earlier studies, the highly evolved *gp85* gene is capable of interacting with receptors, which is crucial for viral entry [18, 32]. Furthermore, the ALV-J-gp85 quality sequencing is typically utilized for sub-atomic study of disease transmission and quasi species variety exhibit as it is the most definitive quality that anyone could hope to find in the GenBank data set [60, 65]. Our two ALV-J isolates’ gp85 gene was sequenced, and the results were compared to other Genbank-deposited ALV-J referencing sequences. The AlQalyubiya-1-EGYALVJ-env and Newvalley-2-EGYALVJ-env isolates, on the other hand, have the highest homology to Chinese strains, according to phylogenetic analysis. Their nucleotide sequence identity is 100%, 99%, 99%, and 98%, respectively, and their amino acid sequence identity is 100%, 99%, 98%, 97%, 96%, and 96%, respectively. Specifically, our earlier findings may indicate that these two ALV-J isolates may originate from the same origins as China or share common ancestors [66]. This pronounced genetic homology with Chinese strains especially within *gp85* gene strongly suggests the global spread of ALV-J and the ALV-J isolates likely transmitted via imported breeding stock or international trade of poultry products across geographically distant regions. Collectively, these current findings highlight the epidemiological linkages between Egyptian and Chinese ALV-J poultry populations and underscore the need for enhanced molecular surveillance to monitor potential introductions and evolutionary dynamics of this rapidly spreading Avian retrovirus. This reasonably high ALV-J investigation rate may be due to the viral vertical transmission. This statement nearly matches to [26], who mentioned that the nucleotide similarity of their Egyptian isolates ranged from 88 to 94% comparable to Chinese strains. These promising results were consistent with [67], who reported that the genome sequencing of their isolates of hemangioma cases revealed high nucleotide and amino acid identities to Chinese strains. Also, these subsequent results came in accordance with [33]. Furthermore, AlQalyubiya-1-EGYALVJ-env and Newvalley-2-EGYALVJ-env isolates shared nucleotide identity percentages of (94%, 93%, 91%, 94%, 87%,95%), and (92%, 92%, 90%, 92%, 87%, 94%); respectively, and on the amino acid level were (95%, 95%; 92%, 94%, 92%, 94%) and (94%, 94%, 92%, 93%, 91%, 93%); respectively with five mentioned Egyptian isolates and American reference strain. The nucleotide identity of both isolates from Dakahlia and Sharqia governorates against other Egyptian isolates was reported to range from 96% to 100% [24]. Our findings are nearly in line with ALV-J disconnect QL1, ALV-J disengage QL4. More importantly, these results are largely in agreement with those of [68], who found homology of 95.5% of their PK19SA01 isolate to the American reference strain. AlQalyubiya-1-EGYALVJ-env and Newvalley-2-EGYALVJ-env isolates shared 81%, 80% similarity with the United Kingdom prototype strain (HPRS-1003). However [26], clearly showed that the Egyptian ALV-J isolates had an identity percentage of 91.2–91.8%, which was rather similar to HPRS-1003. This was evident from our data as well. Furthermore, according to [68], the PK19FA01 strain’s gp85 gene showed the greatest degree of similarity to HPRS-103, at 97.1%. Importantly, we might speculate that importation or chicken breeding may have brought our ALV-J isolates from China. Collectively, the selection criteria for these two specific isolates for sequencing depend on different factors. First, the two-isolate exhibited strong ALV-J–specific RT-PCR amplification with optimal Ct values, indicating a high viral load. Second, preliminary BLAST analysis of partial *gp85* gene of env fragments revealed a high genetic similarity to contemporary Chinese ALV-J strains. Third, the isolate originated from a commercial broiler flocks with active clinical and production problems, increasing its epidemiological relevance. Fourth, geographic representation was considered, as the isolate originated from AlQalyubiya Governorate (northern of Egypt), a major poultry-dense region in Egypt, and New valley Governorate from Upper Egypt (southern of Egypt).

Furthermore, both isolates have low homology and phylogenetically distant from with ALV-J-HPRS103-EgM/00 (Egyptian strain, ALV-J-II), with a percentage of (88%-89%), This result supports that current ALV-J isolates circulating in Egypt have evolved, likely driven by prolonged local circulation or possible introduction from more recent Asian lineages rather than older one. This genetic divergence highlights the dynamic evolutionary feature of ALV-J and underscores the need for continuous molecular surveillance to monitor emerging variants with potential epidemiological significance.

Even though the samples were collected from different locations and periods, the sequence analysis revealed that our two isolates, AlQalyubiya-1-EGYALVJ-env and Newvalley-2-EGYALVJ-env, shared 97% amino acid similarity and 98% nucleotide identity. Also, Fotouh et al. [24] reported identical findings, stating a nucleotide similarity of his ALV-J isolates of 96%. This phylogenetic alignment highlights the necessity for comprehensive surveillance systems that merge genetic monitoring with ecological tracking of vertical transmission, facilitating early investigation of ALV-J and risk reduction to the poultry industry. As there is no particular treatment or vaccination Schedules available for ALV-J, therefore, in order to reduce ALV-J transmission, control measures relied on the removal of positive cases, management strategies, biosecurity initiatives in the chicken farms, and selecting non-infected breeders for poultry industry.

## Conclusions

It was concluded that our data surprisingly confirmed the presence and genetic characteristics of ALV-J strains with myelocytomatosis circulating in chicken embryos at different localities of Egypt for the first time through immunopathological investigation, and other different genetic approaches. ALV-J causes oncogenic manifestation with a prevalence rate of 15%. Furthermore, the ALV-J *gp85* gene analysis of AlQalyubiya-1-EGYALVJ-env, and Newvalley-2-EGYALVJ-env (PQ119499 - PQ119500, ALV-J-II) is high genetic relatedness to Chinese strains (ALV-J-GD27, ALV-J-GD19, ALV-J-GD31) with nucleotide homology of) 100%, 99%, 99%), (98% (; respectively, and the amino acid homology was (100%, 99%, 98%), (97%, 96%, 96%); respectively. Also, our two isolates shared nucleotide similarity of (87%-94%), and the amino acid similarity was (91%-95%) with previously mentioned Egyptian isolates. This study confirmed that our two ALV-J isolates are not completely similar to the current Egyptian isolates. ALV is still a risk to the local poultry population and is continuously distributed in the breeders, as there are no known immunization programs or medications for ALV. This serves as a reminder to renew the breeders by the elimination of ALV-J positive cases, apply periodic genetic monitoring for all recent strains, and consider the possibility of involving ALV-J viral infection as one of the differentials among associated outbreaks. Importantly, it is currently appropriate to use further full genome sequencing of these local isolates to detect other features as the oncogenicity, pathogenesis, antigenicity, and genetic diversity of these circulating strains.

## Data Availability

Available upon request.
